# The Impact of Resilience, Alexithymia and Subjectively Perceived Helplessness of Myocardial Infarction on the Risk of Posttraumatic Stress

**DOI:** 10.1007/s10880-022-09857-w

**Published:** 2022-02-15

**Authors:** Kevin Kirchner, Hartmut Brauer, Sandra Van der Auwera, Hans Jörgen Grabe

**Affiliations:** 1 Department of Psychiatry and Psychotherapy, University Medicine Greifswald, Ellernholzstraße 1-2, 17489 Greifswald, Mecklenburg-West Pomerania Germany; 2 Department of Cardiological Rehabilitation, KMG Klinik Silbermühle, Plau am See, Mecklenburg-West Pomerania Germany; 3 German Centre for Neurodegenerative Diseases (DZNE), Site Rostock/Greifswald, Mecklenburg-West Pomerania Germany

**Keywords:** Alexithymia, Helplessness, Myocardial infarction, PTSD, Resilience

## Abstract

The aim of this study was to investigate the impact of resilience, alexithymia and the subjectively perceived severity (fear of death, pain intensity, helplessness) of myocardial infarction (MI) on posttraumatic symptom severity (PTSS) after MI. Patients were assessed with the Posttraumatic Diagnostic Scale (PDS), Resilience Scale (RS-11) and Toronto Alexithymia Scale (TAS-20). Subjectively perceived severity of MI was measured with three items on a 10-point Likert scale. To test our hypothesis, we applied Pearson correlations as well as multiple hierarchical linear regression analyses. A higher resilience score was significantly associated with lower (*r* =  − .39, *p* < .001) PTSS. Higher scores of alexithymia (*r* = .38, *p* < .01) and subjectively perceived helplessness (*r* = .42, *p* < .001) were associated with higher PTSS. Multiple hierarchical linear regression analyses revealed that resilience, the TAS-20 subscale difficulty identifying feelings (DIF) and especially subjectively perceived helplessness were independent significant predictors for the PTSS, adjusted *R*^2^ = .29, *F*(5, 102) = 9.57, *p* < .001. Our results suggest that resilience reduces the PTSS whereas alexithymia and subjectively perceived helplessness increase the risk. Especially the subjectively perceived helplessness explains a high degree of variance of PTSS and should be assessed to hindering further mental health burden.

## Introduction

Cardiovascular diseases represent one of the main causes of death worldwide (Vilchinsky et al., 2017). Approximately 805,000 Americans experience a coronary heart attack per year (Virani et al., 2021). The sudden occurrence and experienced danger for life associated with myocardial infarctions (MI) represent a high traumatic stressor which may result in the emergence of acute stress disorder and posttraumatic stress disorder (PTSD) as well as depressive disorders as a long term consequence (Roberge et al., 2010; Singh et al., 2017). Edmondson et al. (2012) reported that the prevalence of PTSD after MI is about 12%.

Thus, it is of utmost importance to identify predictive risk factors that increase the risk of mental maladaptation after MI. In their systematic review for cardiac-disease-induced PTSD (CDI-PTSD) Vilchinsky et al. (2017) classified demographic risk variables (i.e. age, sex, socioeconomic status), objective (i.e. intrusive medical procedures, number of days of hospitalization) and subjectively perceived MI severity (i.e. pain intensity, pain duration, perceived life threat). Interestingly, the subjectively perceived MI severity revealed a stronger impact on the development of PTSD after MI than the objective parameters (Vilchinsky et al., 2017). This notion corresponds well with patient reports about distinctive feelings like fear and anger triggered by the perceived life threat, the loss of control as well as the pain during MI (Hari et al., 2010; Wiedemar et al., 2008).

For instance, Guler et al. (2009) have shown that fear of death increased the risk for developing PTSD threefold. Typically, patients with distinctive fear of death are younger, female and report stronger thorax pain (Albarqouni et al., 2016). Additionally, they suffer from the fear of a repeated infarction or report sleeping problems associated with their anxiety not to wake up anymore. Especially, the subjective feeling of loss of control is experienced as unpleasant (Guler et al., 2009; Hari et al., 2010). Based on clinical case studies of patients with implanted cardio defibrillator (ICD) Peregrinova and Hamann (2018) explained the development of traumatic symptoms after the experience of repeated electric shocks and emphasize the role of the attachment behavioral system. They suggested, that positive attachment experiences (i.e. to get help through medical staff) have a positive effect on disease management, whereas negative experiences (i.e. being alone/isolated or being confronted with overwhelmed helpers) lead to a higher probability of getting traumatized. As an ICD-shock is comparable to a heart attack, it is plausible to assume that the consideration above is also applicable to MI. Furthermore, evidence suggest that the perceived pain intensity during the MI increases the risk of PTSD. These findings are also supported by reported positive correlations between the posttraumatic diagnostic scale (PDS) with fear of death, helplessness and pain intensity (Guler et al., 2009; Hari et al., 2010; Wiedemar et al., 2008). Thus, an impact of the subjectively perceived MI severity on PTSD seems to be very likely.

Despite this, not necessarily every patient develops PTSD after MI. A possible explanation for this observation could be individual protective factors, such as resilience (Meister et al., 2016). Resilience can be described as an optimal adaption process after critical life events or even as the positive outcome after traumatic events (Agaibi & Wilson, 2005; Meister et al., 2016). For instance, studies have shown that resilience reduces the depression symptom severity in individuals exposed to childhood abuse or other traumata (Poole et al., 2017; Schulz et al., 2014; Wingo et al., 2010). Further protective effects were also found for individuals with mild traumatic brain injury (Sullivan et al., 2016) and cancer (Groarke et al., 2020; Hu et al., 2018; Seiler & Jenewein, 2019). This might also apply to the adaptation after MI as a critical life event. Furthermore, evidence suggests that resilience decreases the association between adverse childhood experiences and increased inflammatory processes in adulthood (Gouin et al., 2017) and that resilience is associated with a better physical and mental health-related quality of life one year post MI (Kirchberger et al., 2020). Resilience can thus be considered as a possible protective factor which impacts the connection between the experience of traumatic events and the development of posttraumatic symptoms.

Beside resilience, the identification, the communication and thus, the regulation of feelings and emotions, especially the own feelings and emotions, may be relevant for adaptation and coping after MI (Cameron et al., 2014; Meloni et al., 2016; Taylor et al., 2003). A concept that combines these points is alexithymia. According to Meloni et al. (2016), alexithymia describes the difficulty in identifying and describing emotions and the tendency to minimize emotional experiences and to focus the own attention especially on external stimuli. Several studies reported associations of alexithymia with pain and pain intensity (Aaron et al., 2019; Zeng et al., 2016), social support (Lumley et al., 1996; Saikkonen et al., 2018; Zeng et al., 2016), insecure attachment styles (Romeo et al., 2020), psychosocial stress (Terock et al., 2019), dissociation and PTSD (Grabe et al., 2000; Terock et al., 2016) as well as for hypertension and carotid atherosclerosis (Grabe et al., 2010). Similar to resilience, alexithymia is often considered as personality trait (Bagby et al., 2021; Gao et al., 2015).

In context of MI a recent study by Ledermann et al. (2020) suggests that alexithymia increases the occurrence of posttraumatic stress symptoms after MI. They demonstrated that patients with higher levels on the “difficulties describe feelings (DIF)” subscale show indeed significantly more posttraumatic stress symptoms at 3-month-follow-up than those with lower levels. Preti et al. (2013) hypothesized that people with alexithymia were less likely to estimate and evaluate the danger of MI symptoms due to the limitations in information processing and in describing of feelings. Similar results were found by a study on the impact of alexithymia and secure attachment style on the development of PTSD after MI. The authors suggest that stress triggered by MI increases the likelihood of PTSD through the difficulty to regulate successfully negative feelings and emotions like fear, horror or anger (Gao et al., 2015). In addition, through the misinterpretation of the MI symptoms the hospital admission may be delayed. This time-gap frequently results in more severe heart damages which in turn increases the risk for mental stress (Meloni et al., 2016).

As these results suggest, the ability or inability to perceive and manage negative emotions and feelings could substantially explain the association between subjectively perceived MI severity and posttraumatic symptom severity (PTSS) after MI. Referring to this, resilience should diminish this association, since people with high resilience should be able to better cope with these negative feelings. In contrast, alexithymia would alter the ability of emotion regulation and would increase the risk of maladaptation.

Based on these findings we hypothesize that resilience protects against PTSS in subjects after MI and that alexithymia, in contrast, increases the occurrence of PTSS. Additionally, we want to examine the impact of subjectively perceived MI severity on the occurrence of PTSS and her interplay with resilience and alexithymia. To our knowledge this is the first study which addressed resilience, alexithymia as well as subjectively perceived severity as possible common predictors within the context of MI-induced PTSS.

## Materials and Methods

### Participants and Study Design

The study was conducted in the Department of Cardiological Rehabilitation of the KMG Klinik Silbermühle from 04/2019 to 03/2020. Within this time period one hundred fifteen patients with post-MI condition were recruited. All analyses conducted were performed in accordance with the Declaration of Helsinki, including written informed consent of all participating 115 subjects. The survey and methods of the study were approved by the Institutional Review Board of the University of Greifswald. Participants with the diagnosis of PTSD prior to MI and other preexisting psychiatric diagnoses were excluded (*N* = 7) from the analysis.

### Psychometric Assessment

For our assessment, we used the validated German versions of different self-report questionnaires. Alexithymia was assessed using the Toronto Alexithymia Scale (TAS-20; Bach et al., 1996, cited after Franz et al., 2008). The TAS-20 includes 20-items distributed on the three subscales “difficulty identifying feelings” (DIF), “difficulty describing feelings (DDF) and “external-oriented thinking” (EOT) on a 5-point Likert scale (Franz et al., 2008). Higher scores mean higher alexithymia and could range between 20 and 100 for the TAS-20 sum score. Resilience was measured with the short version of the Resilience Scale (RS-11) originally developed by Wagnild and Young in 1993. In sum, the RS-11 comprises 11 items on a 7-point Likert scale (1 = “disagree”, 7 = “agree”). Thereby higher sum scores correspond to higher resilience and could range between 11 and 77 (Schumacher et al., 2005). To measure the severity of posttraumatic stress symptoms, we chose the PDS due to the reported high internal consistency (Hari et al., 2010; Wiedemar et al., 2008) and retest reliability (Griesel et al., 2006). Participants had to rate 17 items on a 4-point Likert scale between 0 = “not at all” and 4 = “often” within the past month. The sum score ranged from 0 up to 51 with higher scores indicate more severe posttraumatic stress. Referring to Wiedemar et al. (2008), Guler et al. (2009) and Hari et al. (2010) the subjective perception of MI severity was rated on three items on a 10-point Likert scale for (1) *fear of dying* (“During my referral to the hospital, the emergency unit, or the intensive care unit, I was afraid I was dying”; 0 = absolutely not true, 10 = absolutely true), (2) *helplessness* (“When the doctor told me I had a heart attack, I was frightened, felt helpless, and was afraid of losing control of the situation”; 0 = absolutely not true, 10 = absolutely true) and (3) *pain intensity* (“Please indicate how strong your pain was during the heart attack”; 0 = no pain, 10 = intolerable pain). Thereby, the term “event” was replaced with the term “heart attack”.

### Statistical Analysis

Descriptive statistics are reported as *n* and percentage for categorical variables and mean, standard deviation and range for metric variables. Group comparison for males and females were performed using *χ*^2^, Mann–Whitney *U* and *t*-tests depending on the distribution of the variables. Assumption of normal distribution and homoscedasticity was checked with Shapiro–Wilk and Levene tests. After descriptive statistics we conducted Pearson correlation analysis for all psychometric variables; PDS, RS-11, TAS-20 and his subscales (DDF, DIF, EOT) as well as for the subjectively perceived MI severity (fear of death, helplessness and pain intensity) to explore their relationships. Correlation coefficients with values of .1, .3 and .5 indicating small, medium and large effect sizes, respectively (Cohen, 1988). *p*-Values were Bonferroni corrected for multiple testing. Based on the correlation results, we identified variable block with high pairwise correlation. To analyze their predictive role on PTSS as well as their interplay, we applied multiple hierarchical linear regression analyses. We set age and sex as control variables and all variables within one identified correlation block as predictor variables. The significance level was set at *p* < 0.05 (two tailed). Data were analyzed using the open source software R (R Core Team, 2020).

## Results

### Sample Characteristics

The total sample size with fully available data comprised *N* = 108 patients with a mean age of 58.4 years (SD = 8.6). 79.6% were men (*n* = 86) and 89.8 (*n* = 97) experienced their first heart attack. Most of the patients were married (62.1%). Besides MI, 75% and 76.9% of patients had a diagnosis of diabetes (*n* = 81) or hypolipoproteinemia (*n* = 83) and 86.1% (*n* = 93) were diagnosed with hypertension. We also found a significant difference for men and women for the occurrence of diabetes, 80.2% vs. 54.5%; *χ*^2^ (1, *n* = 108) = 4.87, *p* = .02. Thus, the sample showed a broad cardio-metabolic risk profile. Despite, no further significant sex differences could be observed. More detailed sample characteristics and results of sex comparisons are shown in Table 1.

**Table 1:** Sociodemographic characteristics (*N* = 108); group comparisons male/female

Parameter	All	Male (*n* = 86)	Female (*n* = 22)	*p* Value of comparison
Age (years)	58.4 ± 8.6	58.4 ± 8.2	58.3 ± 10.5	.58^b^
BMI	28.7 ± 4.5	28.8 ± 4.6	28.3 ± 4.4	.68^c^
PDS	8.7 ± 7.4	8.3 ± 7.3	10.9 ± 7.7	.10^b^
RS	63.0 ± 9.7	63.4 ± 9.5	61.3 ± 10.8	.39^b^
TAS	45.3 ± 10.8	45.0 ± 11.0	46.3 ± 10.6	.61^c^
DDF	12.1 ± 3.6	12.0 ± 3.7	12.2 ± 3.3	.53^b^
DIF	13.7 ± 4.9	13.4 ± 4.9	14.9 ± 4.6	.13^b^
EOT	19.5 ± 4.3	19.6 ± 4.3	19.1 ± 4.4	.94^b^
FoD	3.7 ± 2.9	3.5 ± 2.8	4.3 ± 3.2	.31^b^
HLP	4.2 ± 3.1	4.1 ± 3.1	4.9 ± 3.1	.21^b^
PI	6.1 ± 2.7	6.1 ± 2.6	6.4 ± 2.9	.57^b^
Marital status				
Married	67 (62.1)	55 (64.0)	12 (54.5)	**.04**^**a**^
Divorced	17 (15.7)	14 (16.3)	3 (13.6)	
Widowed/Single	24 (22.2)	17 (19.7)	7 (31.9)	
Hypertension	93 (86.1)	74 (86.0)	19 (86.4)	> .99^a^
Hypolipoproteinemia	83 (76.9)	66 (76.7)	17 (77.3)	> .99^a^
Diabetes	81 (75.0)	69 (80.2)	12 (54.5)	**.02**^**a**^
First heart attack	97 (89.8)	78 (90.7)	19 (86.4)	.70^a^

Data are presented as mean ± SD and *n* (%)

*BMI* body mass index, *DDF* difficulty describing feelings (TAS subscale), *DIF* difficulty identifying feelings (TAS subscale), *EOT* external-oriented thinking (TAS subscale), *FoD* Fear of Death, *HLP* Helplessness, *PI* Pain Intensity, *PDS* Posttraumatic Diagnostic Scale, *RS* Resilience Scale, *TAS* Toronto Alexithymia Scale sum score

*p < .05, **p < .01, ***p < .001

^a^*χ*^2^ Test

^b^*U*-test

^c^*t*-test

### Results of Correlation Analysis

Correlation analysis for the PTSS with the predictors showed significant associations for resilience (*r* =  − .39, *p* < .001), TAS-20 sum score (*r* = .38, *p* < .01), DDF (*r* = .36, *p* < .01), DIF (*r* = .38, *p* < .01) as well as for helplessness (*r* = .42, *p* < .001) and fear of death (*r* = .34, *p* < .01). No associations where found for externally oriented thinking (*r* = .23, *p* > .05) and pain intensity (*r* = .06, *p* > .05). Within the predictors we observed a significant negative association for resilience and alexithymia sum scores (*r* =  − .54, *p* < .001). Both were not associated with fear of death, helplessness and pain intensity which however show strong associations with each other. Altogether, pairwise correlation results revealed a correlation block for resilience and alexithymia as well as for fear of death, pain intensity and helplessness (Table 2).

**Table 2:** Pearson correlation matrix of the dependent and independent variables

Parameters	PDS	RS	TAS	[DDF]	[DIF]	[EOT]	FoD	HLP	PI
PDS	1	–	–	–	–	–	–	–	–
RS	− .39***	1	–	–	–	–	–	–	–
TAS	.38**	− .54***	1	–	–	–	–	–	–
[DDF]	.36**	− .47***	.82***	1	–	–	–	–	–
[DIF]	.38**	− .43***	.84***	.59***	1	–	–	–	–
[EOT]	.23	− .45***	.82***	.56***	.49***	1	–	–	–
FoD	.34**	− .06	.16	.21	.14	.09	1	–	–
HLP	.42***	− .22	.28	.28	.22	.21	.60***	1	–
PI	.06	.17	− .03	− .04	− .04	< .01	.33^*^	.27	1

*DDF* difficulty describing feelings (TAS subscale), *DIF* difficulty identifying feelings (TAS subscale), *EOT* external-oriented thinking (TAS subscale), *FoD* Fear of Death, *HLP* Helplessness, *PI* Pain Intensity, *PDS* Posttraumatic Diagnostic Scale, *RS* Resilience Scale, *TAS* Toronto Alexithymia Scale sum score

*p < .05, **p < .01, ***p < .001. *p* Value adjusted with Bonferroni

### Association Among Resilience, Alexithymia, Subjectively Perceived MI Severity and PTSS

To clarify the associations among the identified correlation blocks and PTSS, we performed multiple hierarchical linear regression analyses (Table 3). In our basic model we added sex and age as control variables (model 1). In a second step, we tested the common predictive influence of resilience and alexithymia (model 2) as they were highly correlated. In a third model we tested the common predictive effect of the subjective measures (perceived fear of death, pain intensity and helplessness) (model 3). In a final model we included all predictor variables that were significant in models 2 and 3 in a final model (model 4). In model 2 both resilience, *β* =  − .26, *t*(103) =  − 2.48, *p* = .01, and alexithymia, *β* = .24, *t*(103) = 2.28, *p* = .03, were significantly related to PTSS. However, in model 3 only subjectively perceived helplessness, *β* = .35, *t*(102) = 3.13, *p* = .002 revealed a significant effect on PTSS. Both models were highly significant and explained about 17% of the variance of PTSS, model 2: adjusted *R*^2^ = .17, *F*(4, 103) = 6.77, *p* < .001; model 3: adjusted *R*^2^ = .18, *F*(5, 102) = 5.43, *p* < .001. Based on the results of models 2 and 3, we tested one further model which included resilience, alexithymia and subjectively perceived helplessness (model 4). In this model the explained variance for PTSS increased to an adjusted *R*^2^ = .27, *F*(5, 102) = 8.94, *p* < .001. Although the impact of resilience, *β* =  − .24, *t*(102) =  − 2.39, *p* = .02 remained stable after adding subjectively perceived helplessness, *β* = .33, *t*(102) = 3.76, *p* < .001, the impact of alexithymia, *β* = .16, *t*(102) = 1.60, *p* = .11 was reduced and not statistically significant anymore. Thus, our data suggest that subjectively perceived helplessness seems to have a greater impact on the occurrence of PTSS after MI than alexithymia and resilience.

**Table 3:** Regression models of posttraumatic symptom severity (PDS score)

	*β*	*p* Value	*R*^2^	Adjusted *R*^2^	Δ*R*^2^	*p* Value
Model 1						
Sex	− .14	.14	.02	< .01		.33
Age	.01	.91				
Model 2						
Sex	− .11	.22	.21	.18	.19	< .001***
Age	.04	.63				
RS	− .26	.01*				
TAS	.24	.03*				
Model 3						
Sex	− .09	.31	.21	.17	.19	< .001***
Age	.09	.29				
FoD	.16	.17				
PI	− .08	.39				
HLP	.35	.002**				
Model 4						
Sex	− .08	.36	.30	.27	.28	< .001***
Age	.11	.20				
RS	− .24	.02*				
TAS	.16	.11				
HLP	.33	< .001***				
Model 5						
Sex	− .06	.49	.32	.29	.30	< .001***
Age	.11	.19				
RS	− .24	.01*				
DIF	.20	.03*				
HLP	.34	< .001***				

*ΔR*^*2*^ comparison with model 1, *DIF* difficulty identifying feelings (TAS subscale), *FoD* Fear of Death, *HLP* Helplessness, *PI* Pain Intensity, *RS* Resilience Scale, *TAS* Toronto Alexithymia Scale sum score

*p < .05, **p < .01, ***p < .001

### Sensitivity Analysis

As Luminet et al. (2021b) propose, the consideration of single alexithymia facets can help to develop a better understanding for underlying processes and how these might influence individual mental and somatic vulnerabilities. Parallel to model 2 we calculated a model where we replace the TAS-20 sum score with the three individual subscales (DDF, DIF, EOT) as predictors. As only the DIF subscale remained significant, we calculated a model 5 parallel to model 4 where resilience, DIF and subjectively perceived helplessness were added as predictors. As regressions results suggest, model 5 explained 29% of variance, a little more than the original model 4. Further, our data showed the good suitability of resilience, *β* = .07, *t*(102) =  − 2.58, *p* = .01, DIF, *β* = .14, *t*(102) = 2.19, *p* = .03 and helplessness, *β* = .21, *t*(102) = 3.88, *p* < .001 as predictors of PTSS after MI. Thereby, especially the subjectively perceived helplessness seems to substantially predict the occurrence of posttraumatic symptoms after MI.

## Discussion

The aim of this study was to explore the common impact of resilience, alexithymia and subjectively perceived fear of death, helplessness and pain intensity on PTSS. Our cardiac sample consisted of relatively young patients (*M* = 58.4 years old) and was strongly dominated by men. Our results suggest that higher alexithymia scores were associated with a higher PTSS whereas higher resilience scores were associated with lower severity. We further found, that patients with greater fear of death and feeling more helpless, showed a greater PTSS. These observations were confirmed by regression results and support the consideration of resilience, alexithymia and, especially, subjectively perceived helplessness as adequate predictors for PTSS. In a joint model we discovered the high influence of subjectively perceived helplessness even after controlling for the effect of resilience and alexithymia. Initially, after adjusting for sex and age, resilience and alexithymia explained 18% of variance of PTSS together and indicated a protective effect of resilience and reinforcing effect of alexithymia. After adding the subjectively perceived helplessness, the three predictors even explained altogether 29% of variance of PTSS. Thereby, subjectively perceived helplessness diminished the influence of alexithymia and revealed their great predictive potential. Although similar patterns were already described by Meister et al. (2016) for resilience and by Gao et al. (2015) for alexithymia, to our knowledge no study has addressed resilience, alexithymia as well as subjectively perceived severity as possible common predictors within the context of MI-induced PTSS so far.

We assume, that people with high resilience scores more often rely on their own skills and thus are capable to cope better with negative feelings triggered by MI. This is also supported by the observation that people with high resilience scores reported less frequently to have felt helpless. As studies of cognitive–emotional processing in alexithymia suggest, low verbal representations of emotions might lead to restricted attentional and memory processes which makes it difficult to connect to external and sensory experiences. Additionally, the externally orienting thinking style leads to connection problems of feelings with thoughts, memories and personal aims (Luminet et al., 2021a). In context of MI, people with high alexithymia scores are thus not able to manage and report their own feelings and probably interpret and evaluate the symptoms wrong. This perhaps, leads to a delayed ask or search for help, i.e. call an ambulance or visit an emergency department and results in a delayed medical treatment with an increased risk for later severe heart damages and posttraumatic symptoms (Meloni et al., 2016). This notion is also supported by the observed none-significant positive associations of DDF and DIF with fear of death and helplessness (Table 2). Thus, alexithymia might impact the perception of MI and might lead to an increased feeling of helplessness. According to this assumption, helplessness would mediate the link between alexithymia and PTSS. As our limited sample size made it difficult to adequately conduct a mediation analysis, we propose future analyses to investigate a possible mediating effect. Regarding subjectively perceived helplessness, we assume that mainly the feeling of losing control and the associated anxiety might explain the impact on PTSS. Loss of control as a unpleasant state was already described in previous studies (Guler et al., 2009; Hari et al., 2010). From a psychological perspective, the illusion of control among life events is associated with a feeling of safety, especially for people with distinct internal locus of control. Since a MI is such an uncontrollable life event, we propose, that first responders and clinicians try to mediate a feeling of safety and security during the whole treatment process. This might reduce anxiety symptoms as well as the feeling to lose the control and, thus, the feeling of helplessness which in turn decrease the occurrence of PTSS in long term.

Since resilience and alexithymia are mainly described as personality traits, it remains unclear whether people with high alexithymia scores show generally lower resilience scores or vice versa. However, in some papers resilience is also considered more as a state or ability, respectively, than a trait (Meister et al., 2016). If so, the appropriation of new coping skills should also be feasible for people with high alexithymia scores. The high negative association might also be traceable on the differences in cognitive–emotional processing. As regression results out of our sensitivity analysis showed, substantially the DIF subscale contributes a significant amount of variance additionally to resilience and subjectively perceived helplessness. This finding supports several previous studies. It was reported, that DIF reinforces the association between acute stress symptoms and posttraumatic stress symptoms after MI (Ledermann et al., 2020). Likewise, a cross sectional survey to the roles of attachment, alexithymia and PTSD in patients with MI observed, although DDF and DIF were significantly correlated with PTSS, that only DIF was a significant predictor (Gao et al., 2015). As this evidence suggest, the missing ability to identify feelings might increase PTSS after MI.

Surprisingly and in contrast to previous studies (Hari et al., 2010; Wiedemar et al., 2008) we found no significant correlation for pain intensity and PTSS. Similar results were reported by Guler et al. (2009). There are two possible explanations. Either our sample did not experience strong pain during the MI or its impact is not as high as suggested.

There are a number of limitations to this study. First, due to the cross sectional study design we are not able to derive causal relationships as we are not aware of the RS-11 and TAS-20 score before the MI of the patients. Although alexithymia can be considered as a stable trait (*r*_tt_ = .71–.81 for different languages, Kooiman et al., 2002), MI triggered alterations in the amount of alexithymia cannot be excluded. For example, studies reported an increase in neuroticism following the experience of a negative life event (Jayawickreme et al., 2021). Also, trait alexithymia might have been influenced in this way. Second, we are not able to exclude selection effects due to a small sample size and self-selection (a total of *n* = 129 patients decline a participation). Despite these limitations our study provides useful insights into the relationship between resilience, alexithymia and PTSS after MI.

Altogether, our study provides strong evidence for the assumption that resilience, DIF and subjectively perceived helplessness are adequate predictors for PTSS after MI. A novel contribution of the current study was the exploration of psychological resilience as a protective and alexithymia and as a vulnerable factor for PTSS and their interplay with subjectively perceived severity among individuals after MI. Surprisingly, especially subjectively perceived helplessness explained a high proportion of variance. In context of alexithymia, particular the TAS-20 subscale DIF predicted the PTSS well. Thus, our study highlights the importance for clinicians to identify low resilient, high alexithymic as well as patients who felt helpless after MI, to prevent them against further health burdens. Clinically, the current findings include important implications for treatments, especially psychological, and the future research. For instance, psychological interventions could reduce problems in cognitive–emotional processing and prevent patients from further physical and mental health damage due the improvement of coping strategies and the imparting of strategies to identify, explain and manage negative feelings and emotions triggered by MI. Further, providing a safe and secure surrounding during the treatment and improvement of patient self-efficacy might decrease the feeling of anxiety, loss of control and helplessness. Thus, these strategies could reduce the occurrence of acute stress disorders or PTSD in long term. In addition, we propose for future research the recruitment of a substantially larger sample size. Also, resilience, alexithymia and particularly subjectively perceived helplessness should be considered in the development of a possible screening instrument for clinicians.

## Data Availability

Due to data protection law it is not possible to share this clinical data and material.
